# Surface ozone pollution in China: Trends, exposure risks, and drivers

**DOI:** 10.3389/fpubh.2023.1131753

**Published:** 2023-03-21

**Authors:** Chao He, Qian Wu, Bin Li, Jianhua Liu, Xi Gong, Lu Zhang

**Affiliations:** ^1^College of Resources and Environment, Yangtze University, Wuhan, China; ^2^School of Resource and Environmental Sciences, Wuhan University, Wuhan, China; ^3^School of Low Carbon Economics, Hubei University of Economics, Wuhan, China; ^4^Collaborative Innovation Center for Emissions Trading System Co-constructed by the Province and Ministry, Wuhan, China; ^5^State Key Laboratory of Freshwater Ecology and Biotechnology, Institute of Hydrobiology, Chinese Academy of Sciences, Wuhan, China

**Keywords:** surface ozone, spatial-temporal pattern, exposure risks, health risks, dominant drivers

## Abstract

**Introduction:**

Within the context of the yearly improvement of particulate matter (PM) pollution in Chinese cities, Surface ozone (O_3_) concentrations are increasing instead of decreasing and are becoming the second most important air pollutant after PM. Long-term exposure to high concentrations of O_3_ can have adverse effects on human health. In-depth investigation of the spatiotemporal patterns, exposure risks, and drivers of O_3_ is relevant for assessing the future health burden of O_3_ pollution and implementing air pollution control policies in China.

**Methods:**

Based on high-resolution O_3_ concentration reanalysis data, we investigated the spatial and temporal patterns, population exposure risks, and dominant drivers of O_3_ pollution in China from 2013 to 2018 utilizing trend analysis methods, spatial clustering models, exposure-response functions, and multi-scale geographically weighted regression models (MGWR).

**Results:**

The results show that the annual average O_3_ concentration in China increased significantly at a rate of 1.84 μg/m^3^/year from 2013 to 2018 (160 μg/m^3^) in China increased from 1.2% in 2013 to 28.9% in 2018, and over 20,000 people suffered premature death from respiratory diseases attributed to O_3_ exposure each year. Thus, the sustained increase in O_3_ concentrations in China is an important factor contributing to the increasing threat to human health. Furthermore, the results of spatial regression models indicate that population, the share of secondary industry in GDP, NOx emissions, temperature, average wind speed, and relative humidity are important determinants of O_3_ concentration variation and significant spatial differences are observed.

**Discussion:**

The spatial differences of drivers result in the spatial heterogeneity of O_3_ concentration and exposure risks in China. Therefore, the O_3_ control policies adapted to various regions should be formulated in the future O_3_ regulation process in China.

## 1. Introduction

Within the context of the yearly improvement of particulate matter (PM) pollution in Chinese cities, O_3_ concentrations are increasing instead of decreasing and are becoming the second most important air pollutant after PM (1). According to the data published by the China General Environmental Monitoring Station, the daily maximum hourly average 90th percentile concentration of O_3_ in 338 prefecture-level cities in China increased from 140.0 μg/m^3^ in 2014 to 151.0 μg/m^3^ in 2018, and the number of days exceeding the standard increased from 6.1% in 2014 to 8.4% in 2018, and the O_3_ concentration in some regions has exceeded the secondary concentration limit (160 μg/m^3^) for air quality in China (2). Long-term exposure to high O_3_ concentrations not only affects urban air quality (3), damages human health (4), reduces food production (5), affects atmospheric radiation balance (6), and even influences global climate change (7). Due to its importance to the atmospheric environment and climate change, O_3_ has received continuous attention from the scientific community and relevant regulatory administrations in the past decades.

To deeply understand the O_3_ pollution in China, a large number of researchers have conducted extensive investigations on O_3_ pollution levels, spatial and temporal patterns, trends, exposure risks, and drivers in China from different spatial and temporal scales over the past decade (8–10). For example, Gong et al. (11) revealed the dominant meteorological controls on surface O_3_ pollution in 16 Chinese cities from 2014 to 2016 using a generalized additive model (GAM); Cao et al. (12) studied the spatial and temporal patterns of O_3_ pollution and ecological risks in the rainfed area of West China, Southwest China, based on ground-based data. Zhan et al. (2) estimated the health risk due to O_3_ pollution in the Yangtze River Delta (YRD) region between 2015 and 2019 based on the exposure-response function, and their results showed that the population of premature respiratory deaths due to O_3_ pollution was 5,889 cases per year from 2015 to 2019, and found that the population of premature deaths was extremely sensitive to O_3_ pollution. In addition, Gao et al. (3), Maji et al. (13), and Lu et al. (14) also performed relevant studies on health risks due to O_3_ pollution in China from different regions.

The numerous studies mentioned above are important references for a comprehensive assessment of the O_3_ pollution in China, but these studies still have the following shortcomings. First, there is significant spatial heterogeneity in surface O_3_ pollution, with a few individual cities or regions of O_3_ pollution not being a substitute for the level of O_3_ pollution in China. Second, there are potential spatial associations between exposure risk and health risk of populations to surface O_3_ pollution, and unfortunately, previous studies have tended to ignore their interrelationships. Third, the effects of drivers on O_3_ concentrations are spatially variable, and previous studies have tended to focus on the combined effects of drivers on O_3_, neglecting the spatial and temporal differences in the effects of drivers on O_3_ concentrations.

Therefore, the main objectives of this study include: (1) investigating the spatial and temporal patterns and trends of O_3_ concentrations in China using trend analysis and spatial clustering based on a high spatial and temporal resolution O_3_ concentration dataset; (2) examining the spatial and temporal associations of population exposure risk and health risk attributable to O_3_ pollution using population exposure risk models and exposure-response functions; and (3) revealing the drivers of differences in O_3_ distribution in China from a spatial perspective based on a multi-scale geographically weighted regression (MGWR) model. This study has important practical implications for assessing the future health burden caused by O_3_ pollution and its resulting health costs in China; meanwhile, it has important implications for how to equitably allocate healthcare resources and environmental management costs in the future planning and construction of healthy cities and smart cities in China.

## 2. Materials and methods

### 2.1. Study area

**Figure 1:**
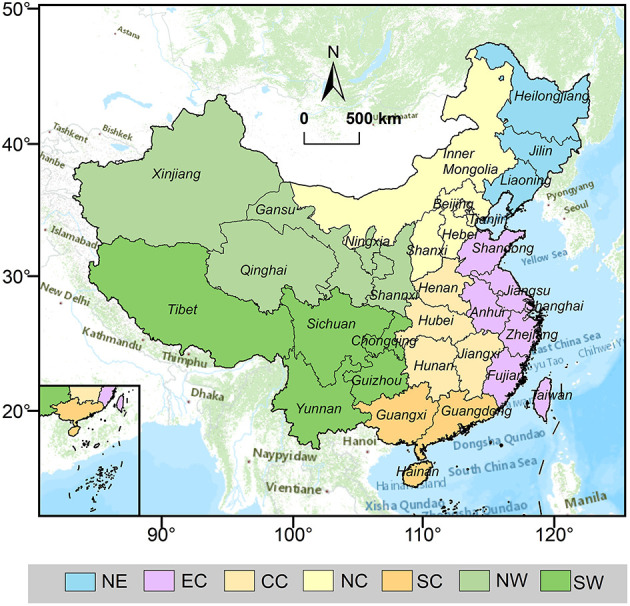
Study area spatial distribution map.

This study focuses on China, including 31 provinces in the Chinese mainland, excluding Hongkong, Macau, Taiwan, and Hainan. Based on the social, natural, economic, and human environment, these 31 regions were further categorized into seven geo-administrative regions, including North China (Beijing, Tianjin, Hebei, Shanxi, Inner Mongolia), South China (Guangdong, Guangxi, Hainan), East China (Shanghai, Anhui, Fujian, Jiangsu, Jiangxi, Shandong, Zhejiang), Central China (Henan, Hunan, Hubei), Southwest China (Yunnan, Guizhou, Sichuan, Chongqing, Tibet), Northwest China (Shaanxi, Gansu, Ningxia, Qinghai, Xinjiang), and Northeast China (Heilongjiang, Jilin, Liaoning) (Figure 1).

### 2.2. Data source

The daily maximum 8-h O_3_ concentration (MDA8) reanalysis dataset of 10 × 10 km from January 1, 2013, to December 1, 2018, is from the tracking air pollution in China (http://tapdata.org/). The dataset is based on a machine learning algorithm and multi-data information fusion inversion. Its comprehensive construction combines ground monitoring data, satellite remote sensing data, high-resolution emission inventory data, air quality model simulation, and other multi-source data, which greatly improves the spatial and temporal accuracy of the data inversion results compared with the previous air quality reanalysis data (15). The daily O_3_ concentrations in 360 prefecture-level cities in China during the study period were obtained from the China National Environmental Monitoring Center (http://www.cnemc.cn/sssj/). In order to reduce the error in the calculation of the health risk model, we calculated the 90th percentile concentration of the MDA8 O_3_ concentration from the interannual scale based on the daily MDA8 O_3_ concentration as the threshold.

The population size (Pop), the proportion of secondary industry to GDP (S_GDP), disposable income per capita (P_GDP), and soot emissions (Dust) for 360 prefecture-level cities in China during the study period were obtained from the China Statistical Yearbook (http://www.stats.gov.cn/tjsj/ndsj/#). The nitrogen oxide (NOx) and volatile organic compound (VOC) emissions were obtained from the China Multiscale Emissions Inventory Model (http://meicmodel.org/). The 1 × 1 km spatial resolution population data were obtained from the World pop dataset (https://www.worldpop.org/).

The daily meteorological data were obtained from the China Meteorological Data Network (http://data.cma.cn/) during the study period. The meteorological data obtained in this study mainly include air temperature (Tem, °C), sea level pressure (Pa, Pa), relative humidity (Hum, %), 2-m mean wind speed (WS, m/s), 1-h precipitation (Pre, mm), and 10-min mean visibility (Vis, m).

### 2.3. Trend analysis

Trend analysis is usually used for the analysis of temporal dynamics of air pollutants to explore the interannual rate of pollutant changes (16). In this paper, the rate of change of O_3_ concentrations in China from 2013 to 2018 was analyzed based on the trend analysis method, which is calculated as Equation (1):


(1) $$\begin{array}{l} Trend=\frac{n\times \sum_{i=1}^{n}\left(i\times O_{3_{i}}\right)-\left(\sum_{i=1}^{n}i\right)\left(\sum_{i=1}^{n}O_{3_{i}}\right)}{n\times \sum_{i=1}^{n}i^{2}-{\left(\sum_{i=1}^{n}i\right)}^{2}} \end{array}$$

Where O_3_ indicates the O_3_ concentration of each cell; *n* indicates the time span, here the time span is 6; and *i* is the time unit.

### 2.4. Population exposure risk model

Previous studies have shown that significant heterogeneity in the spatial distribution of air quality concentrations and population density leads to major spatial differences in the exposure risks of populations to air quality (17). In addition, health risks due to exposure to pollutants are usually defined as a function of the multiplication of population density and pollutant concentration (18). Although the exposure risk intensity in the area can be quantified to some extent, it cannot distinguish the severity of the local area relative to the whole. To address this issue, we introduced a model for the relative exposure risk of the population attributable to O_3_ exposure, as shown in Equation (2), which can evaluate the exposure status in each pixel of the cells (19):


(2) $$\begin{array}{l} R_{i}=\frac{P_{i}\times C_{i}}{\overset{n}{\underset{i=1}{\sum }}P_{i}\times \frac{C_{i}}{n}} \end{array}$$

where *R*_*i*_ indicates the risk of population exposure in grid cell *i*; *P*_*i*_ indicates the number of exposed populations in grid cell *i*; *C*_*i*_ indicates the O_3_ concentration in grid cell *i*, and *n* indicates the total number of grid cells in the study area. To better reflect the spatial difference of relative population exposure risk, we categorized the population exposure risk as extremely low risk, low risk, lower risk, higher risk, high risk, and extremely high risk by using the reclassification method in ArcGIS10.8 software. The corresponding exposure risk values are *R*_*i*_ = 0, 0 < *R*_*i*_ ≤ 1, 1 < *R*_*i*_ ≤ 2, 2 < *R*_*i*_ ≤ 3, 3 < *R*_*i*_≤ 5 and *R*_*i*_> 5, respectively. A higher value of *R* indicates a higher exposure risk.

### 2.5. Health risk model

In this study, a standard damage function was applied to estimate the population of premature deaths from respiratory diseases due to O_3_ exposure. The specific equations are shown in Equations (3) and (4), and the relationships shown in the following equations have been extensively applied in previous studies (14, 20, 21).


(3) $$RR=\{\begin{array}{c} e^{\beta (x-x_{0})},x>x_{0}\\ 1,x\leq x_{0} \end{array}$$

(4) $$\begin{array}{l} \Delta M\;=\;y_{0}\times Pop\times \left[\left(RR-1\right)/RR\right] \end{array}$$

where *RR* is the relative risk; (*RR*−*1*)/*RR* is the attributable fraction; *x*_*i*_ is the O_3_ concentration in a city *i* or grid *i*; *x*_0_ is the threshold concentration; β is the exposure-response coefficient, which represents the additional health risk associated with an increase in unit O_3_ concentration (22, 23); Δ*M* is the number of premature deaths of respiratory diseases attributable to exposure to the O_3_ environment; *y*_0_ is the baseline mortality rate of respiratory diseases, and *pop* is the number of the exposed population. In this study, the mortality rate of respiratory diseases was obtained from the National Bureau of Statistics, where the crude mortality rate of respiratory diseases *y*_0_ (1/100,000) in urban China from 2013 to 2018 was, 76.61, 74.17, 73.36, 69.03, 67.20 and 68.02, respectively. β values in this study were obtained from Shang et al. (24), per 10 μg/m^3^ with a value of 0.48% (95% CL: 0.38%, 0.58%). Song et al. (25) concluded that the exposure-response coefficients obtained from a meta-analysis by Shang et al. (24) based on a 33–time series and case-crossover study conducted could to some extent reflect the health risks attributed to air pollution in China. Meanwhile, which has widely been used in several past studies for China (26, 27).

### 2.6. Multi-scale geographically weighted regression

Compared with the classical geographically weighted regression model (GWR), the MGWR model was a flexible regression model (28). Each regression coefficient was obtained based on local regression, and the bandwidth is specific. In addition, the GWR model uses weighted least squares in the fitting operation, while the MGWR model was equivalent to a generalized additive model (GAM), which could perform regression analysis on spatial variables with linear or non-linear relationships, and was also an effective tool for dealing with various complex non-linear relationships of spatial variables (29). Assuming that there are *n* observations, for observation *i* ∈ {1,2,3,…, *n*} at location (*U*_*i*_, *V*_*i*_), the MGWR were calculated as follows (30):


(5) $$y_{i}\;=\;\beta_{0}(U_{i},V_{i})+\sum j\beta_{bwj}(U_{i},V_{i})X_{ij}+\varepsilon_{i}$$

where *y*_*i*_ is the response variable O_3_ concentration, β_0_(*U*_*i*_, *V*_*i*_) is the intercept, *X*_*ij*_ is the *j*_*th*_ predictor variable *i*, β_*bwj*_(*U*_*i*_, *V*_*i*_) is the *j*_*th*_ coefficient, *bwj* in β_*bwj*_ indicates the bandwidth used for calibration of the *jth* conditional relationship, ε_*i*_ is the error term. In addition, the spatial kernel function type selected during the model operation is bisquare, the bandwidth search type is golden, and the model parameter initialization type takes GWR estimation as the initial estimation model.

### 2.7. Research framework

**Figure 2:**
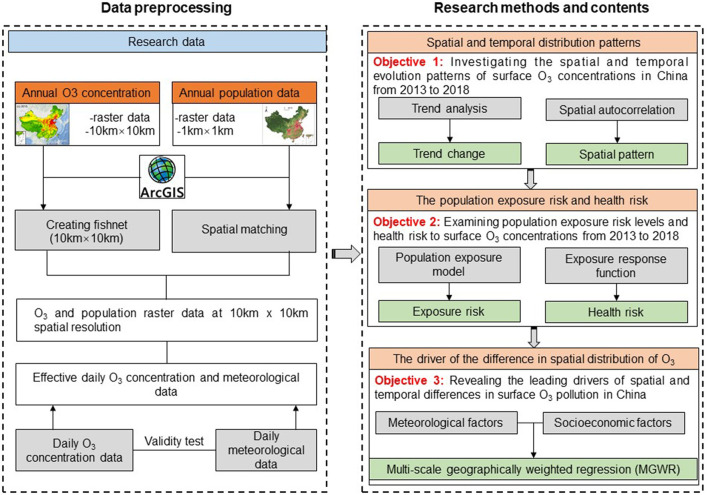
Research framework.

This study used the trend analysis method, spatial autocorrelation model, population exposure risk model, exposure-response function, and MGWR model to analyze the spatial-temporal pattern, exposure risk, health risk, and driving factors of O_3_ concentration in China from 2013 to 2018. Firstly, we use the trend analysis method and spatial autocorrelation model to explore the changing trend and spatial-temporal distribution of O_3_ concentration in China. Secondly, we selected the population exposure risk model and exposure-response function to investigate the population exposure risk and health risk attributed to O_3_ pollution, and discussed their temporal and spatial correlation characteristics. Finally, we use the MGWR model to reveal the dominant factors of spatial distribution difference of O_3_ concentration in China. Additionally, in this study we used O_3_ concentration reanalysis data at 10 × 10 km resolution and population raster data at 1 × 1 km resolution to investigate the exposure risks and health risks attributed to O_3_ pollution. To spatially match the 10 × 10 km O_3_ concentration reanalysis data, we used the aggregation module of ArcGIS10.6 software to quantitatively change the spatial resolution of the 1 km × 1 km population data. During the aggregation calculation, the output image element cell size was set to 10 × 10 km, i.e., 0.01° × 0.01°, and the nearest neighbor assignment method was selected for the aggregation technique. Figure 2 shows the research framework of this paper.

## 3. Results

### 3.1. Spatial and temporal distribution patterns

**Figure 3:**
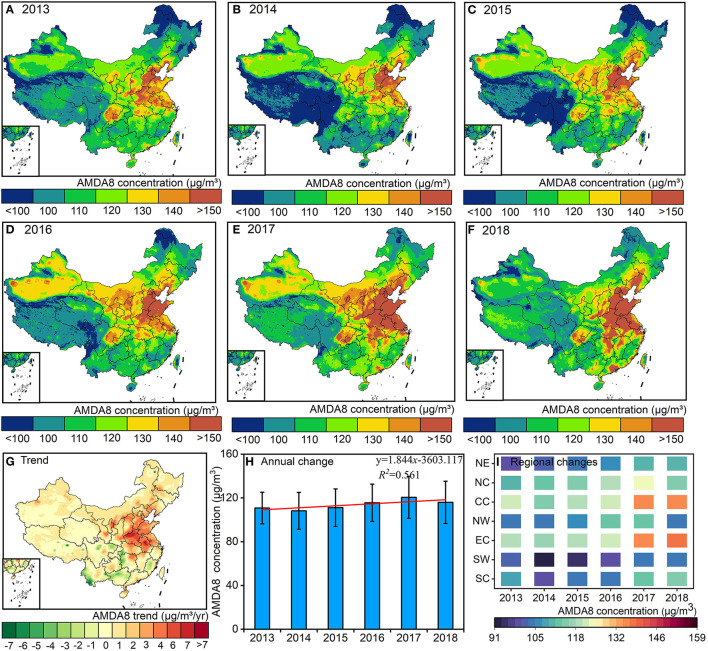
The spatial distribution and trend of O_3_ concentration from 2013 to 2018. Where, **(A–F)** represents the spatial distribution of O_3_ concentration; **(G)** represents the trend change of O_3_ concentration; **(H)** represents the annual average O_3_ concentration variation; **(I)** shows the annual average O_3_ concentration change in different regions of China.

Figure 3 shows the temporal and spatial distribution and changing trend of the annual average concentration of MDA8 (AMDA8, O_3_) from 2013 to 2017 in China. From 2013 to 2018, the annual average O_3_ concentrations in China were 110.75, 108.21, 111.13, 115.57, 120.49, and 115.95 μg/m^3^, respectively, and changed at a rate of 1.84 μg/m^3^/yr increase (Figure 3H). From a spatial and temporal perspective, the highest annual average O_3_ concentrations were found in central China in 2013, 2015, and 2016, with annual average O_3_ concentrations of 121.87, 118.84, and 122.78 μg/m^3^, respectively. The highest annual average O_3_ concentrations in 2014, 2017, and 2018 were all found in East China, with annual average O_3_ concentrations of 116.98, 135.03, and 137.91 μg/m^3^, respectively. In comparison, the lowest O_3_ concentration in 2013 occurred in the Northeast region (98.33 μg/m^3^), the lowest O_3_ concentrations from 2014 to 2017 occurred in the Southwest region of China (90.86, 94.43, 99.20, and 104.27 μg/m^3^), and the lowest O_3_ concentration in 2018 occurred in the Northwest region (103.44 μg/m^3^) (Figures 3A–G). Since 2013, 89.62% of China's territory has experienced a significant increase in annual average O_3_ concentrations, with 2.73% of the regions experiencing an average rate of change in annual average O_3_ concentrations exceeding 5.00 μg/m^3^/yr. However, the rate of variation of O_3_ concentration varies from region to region has strong spatial variability. The rate of change of O_3_ concentration in the Central Plains urban agglomeration is the most variable in terms of the country, with its O_3_ concentration change rate exceeding 4.0 μg/m^3^/yr. In contrast, the rate of change of O_3_ concentration in the Chengdu-Chongqing urban agglomeration (−0.3 ± 1.0 μg/m^3^/yr), Southwest China (−0.5 ± 1.1 μg/m^3^/yr) and South China (−1.0 ± 1.4 μg/m^3^/yr) decreases significantly (Figure 3G).

**Figure 4:**
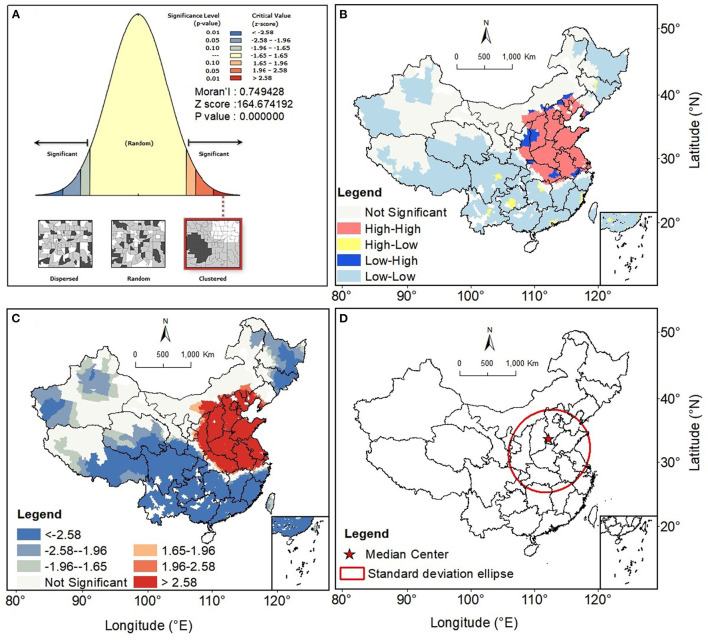
Spatial clustering characteristics of the rate of change of O_3_ concentration in county-level units in China, 2013–2018. **(A)** Global spatial autocorrelation test results; **(B)** Spatial distribution of the spatial clustering of O_3_ concentration variation rates; **(C)** Spatial distribution of cold and hot spots for the rate of change of O_3_ concentration, in the cold and hot spot analysis we used Getis Ord Gi* analysis to calculate *Z* scores, where |*Z*scores| > 1.65 corresponds to *p* < 0.10, |*Z*scores| > 1.96 corresponds to *p* < 0.05, |*Z*scores| > 2.58 corresponds to *p* < 0.01. *Z* scores are negative indicating a cold spot, and a positive *Z* score indicates a hot spot; **(D)** Spatial distribution of the standard ellipse of the rate of variation of surface O_3_ concentration and the center of change in China from 2013 to 2018.

Figure 4 represents the spatial clustering characteristics of the rate of variation of O_3_ concentration at county-level units in China from 2013 to 2018. The results show that the global Moran's *I* index is significant at the 1% level, indicating a consistent and enhanced positive spatial autocorrelation in the rate of variation of O_3_ concentration (Figure 4A). The results of the hot spot analysis show that there is a significant hot spot (HH) region for O_3_ concentration growth rate, which is mainly contiguous and focused in Shaanxi, Shanxi, central Inner Mongolia, Beijing–Tianjin–Hebei (BTH), southwest Liaoning, central Henan, eastern Hubei, Anhui, Jiangsu, and Shandong in China, which are the regions with the strongest O_3_ growth rate in China. In addition, we found a significant cold spot area (LL) covering a large part of China (about 90% of the territory). These regions are mainly located in northeastern, southern, southwestern, eastern, and northwestern China, where the growth rate of O_3_ concentration is relatively low and even decreasing regions are observed (Figures 4B, C). The standard deviation ellipsometric analysis evaluated the overall variations in the spatial pattern of O_3_ concentration growth rate from 2013 to 2018 in China (Figure 4D). It can be found that the regions with significantly increased O_3_ concentration growth rates are mainly concentrated in BTH, Shanxi, Shandong, Jiangsu, Jiangxi, Anhui, Hubei, Henan, and Shaanxi in China. This result also indicates that the above-mentioned regions are the primary contributors of O_3_ during the whole study period in China. Meanwhile, the center of the median growth rate of O_3_ concentration is located north of the standard deviation ellipse arithmetic center, indicating that the growth rate of surface O_3_ concentration is greater in northern China than in southern China.

### 3.2. The population exposure risk and health risk

**Figure 5:**
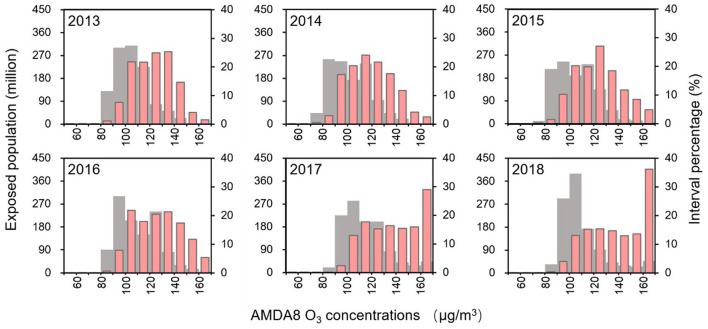
Probability distribution of the total population exposed to different O_3_ concentrations from 2013 to 2018. The red and gray bars indicate the total population and the proportion of the population exposed to different O_3_ concentrations, respectively.

**Figure 6:**
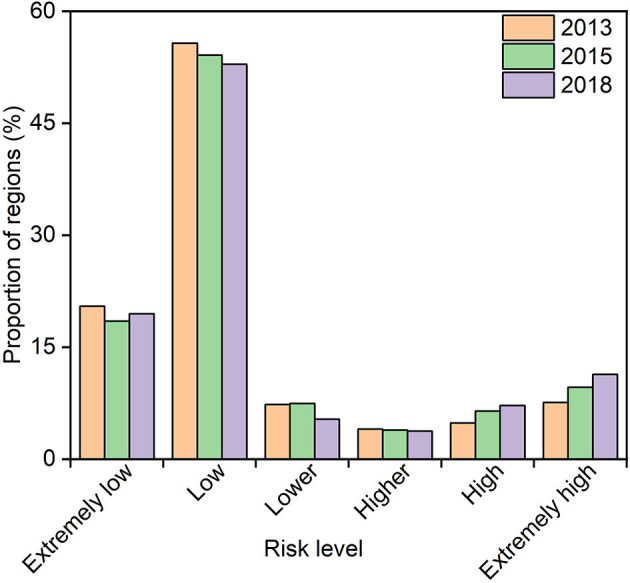
Proportion of regions with different O_3_ exposure risk levels to the total land area (%).

**Figure 7:**
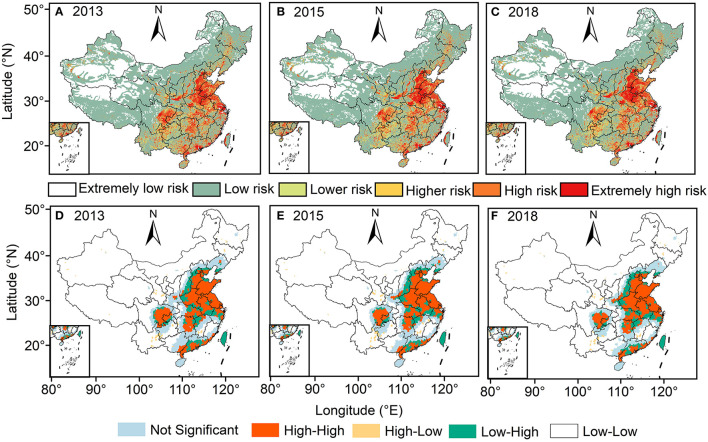
Spatial distribution pattern of population exposure risk levels attributed to O_3_ pollution from 2013 to 2018. **(A–C)** Indicates the spatial distribution of population exposure risk levels in 2013, 2015, and 2018, respectively; **(D–F)** Indicates the spatial clustering of population exposure risk levels in 2013, 2015, and 2018, respectively.

Overall, the total population exposed to O_3_ > 160 μg/m^3^ increased from 1.2% in 2013 to 28.9% in 2018, compared to a decrease in the population exposed to O_3_ < 160 μg/m^3^ from 7.2% in 2013 to 3.6% in 2018 (Figure 5). Figures 6, 7 represents the spatial pattern of exposure risk levels attributed to O_3_ pollution in 2013, 2015, and 2018. We found that most regions have remained at low (52.89–55.73%) or extremely low (19.48–20.48%) O_3_ exposure risk levels over three time periods in China. From a temporal perspective, only 4.83% of the territory of the country was at high exposure to O_3_ pollution in 2013, and this percentage increased to 6.45 and 7.19% in 2015 and 2018, respectively. Similarly, the area of the territory exposed to extremely high risk also exhibits a marked increasing trend, from 7.61% in 2013 to 9.62% in 2015 and further to 11.35% in 2018 (Figures 7A–C). Spatially, the distribution patterns of O_3_ exposure risk levels were similar for the three time periods of 2013, 2015, and 2018 in China. With the rapid increase of O_3_ concentration in the North China Plain, the high exposure risk level regions of BTH and YRD have been continuous, which constitute a high O_3_ exposure risk level aggregation area including the Bohai Rim, YRD, Pearl River Delta (PRD), Shanxi and Guanzhong Plain urban clusters. Spatially, the distribution patterns of O_3_ exposure risk levels were similar for the three time periods of 2013, 2015, and 2018 in China. In contrast, the extremely low risk and lower risk areas of O_3_ pollution are widely distributed in China, which is mainly located in most regions of northwest, southwest, and northeast in China (Figures 7D–F).

**Figure 8:**
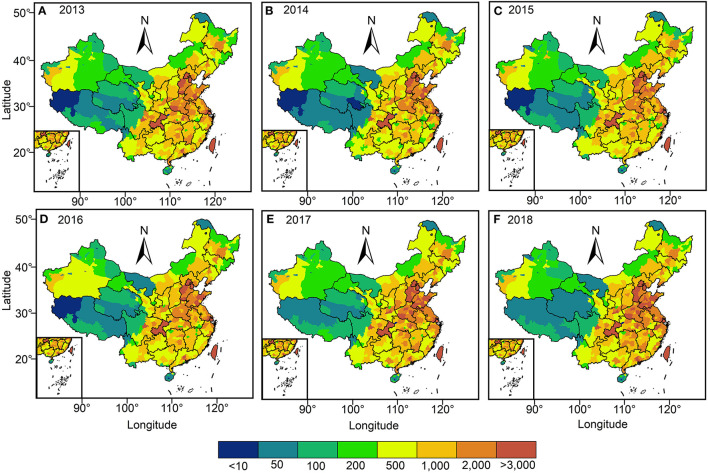
Spatial distribution of premature deaths attributable to O_3_ exposure in China, 2013 to 2018. **(A–F)** indicates the spatial distribution of premature death population attributable to O_3_ exposure at the prefecture-level city scale from 2013 to 2018 in China, respectively.

Figure 8 indicates the spatial and temporal distribution of premature deaths from respiratory diseases attributable to O_3_ exposure from 2013 to 2018. Overall, there was an average of over 24,000 premature deaths from respiratory diseases due to O_3_ exposure per year in China from 2013 to 2018, and the growth rate fluctuated at 1,178 cases per year (*p* < 0.05). Specifically, the number of premature deaths attributable to O_3_ exposure increased from 236,200 in 2013 to 272,300 in 2018, an increase of 36,100 cases compared to 2013. Spatially, the regions with <500 cases of premature death due to O_3_ exposure are mainly located in Tibet, Qinghai, east of Xinjiang, west of Sichuan, west of Inner Mongolia, Liaoning, and Heilongjiang; the regions with more than 500 cases are mainly located in the region east of Hu line, mainly including most of eastern China and western Xinjiang, central Inner Mongolia, southern Gansu, most of southern China, most of northern China, and Liaoning in northeast China. The regions with more than 1,000 cases are mainly located in BTH, Sichuan–Chongqing region, Fenwei Plain, East China Plain, Jianghan Plain, Yangtze River Delta, and Pearl River Delta region. Meanwhile, the regions with more than 1,000 cases of premature death due to O_3_ exposur e are further expanding over time.

### 3.3. The driver of the difference in the spatial distribution of O_3_

Multicollinearity refers to the distortion of model estimates due to significant correlations between the independent variables in the linear modeling regression process. Therefore, before conducting model regression analysis, to test whether there is multicollinearity between each explanatory variable, we use variance inflation factor (VIF) to test the multicollinearity problem between each explanatory variable, and previous studies have shown that when VIF ≥ 10, it indicates that there is a serious multicollinearity problem between the dependent variable and the independent variable. multicollinearity problem, which should be removed from the actual model operation. The collinearity test in this study was performed in SPSS 25.0 software and the results of the analysis showed that the range of VIF values for all explanatory variables was 1.000–9.765, which indicates that there was no cointegration between the dependent and independent variables. Table 1 indicates the diagnostic information of the MGWR model for the socioeconomic and meteorological factors. In terms of the number of valid parameters, the goodness–of–fit *R*^2^ for the responses of socioeconomic and meteorological factors to O_3_ concentrations are 0.861 and 0.799, respectively, and the residual sum of squares (RSS) is 136.297 and 136.51 μg/m^3^, respectively, with the absolute values of the deficit information criterion (AIC) and the log-likelihood value (Log-likelihood) < 5,000. These regression results indicate that MGWR uses fewer parameters to obtain regression results that are closer to the true values and can be fully used to assess the relationship between O_3_ pollution and socioeconomic and meteorological factors.

**Table 1:** Diagnostic information of MGWR model.

**Evaluation indicators**	**Socio-economic factors**	**Meteorological factors**
Residual sum of squares (RSS)	136.297	136.506
Log-likelihood	−423.861	−418.699
Degree of Dependency (DoD)	0.498	0.476
AIC	1,160.521	1,045.466
AICc	1,220.301	1,083.609
BIC	1,925.094	1,515.614
*R^2^*	0.861	0.799
Adj. *R^2^*	0.835	0.763

**Figure 9:**
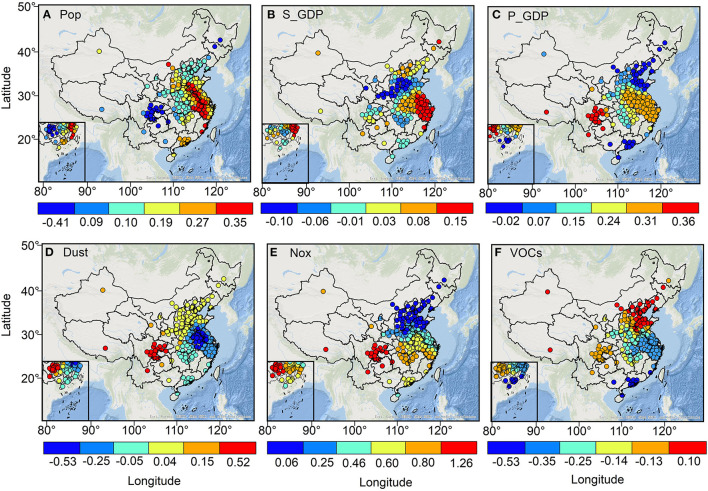
The spatial distribution of regression coefficients for the major socioeconomic factors of Pop **(A)**, S_GDP **(B)**, P_GDP **(C)**, Dust **(D)**, NOx **(E)**, and VOCs **(F)**.

Figure 9 indicates the spatial distribution of regression coefficients of socio-economic factors. The high values (>0.27) of regression coefficients for the total population are mainly located in North and East China, where the total population is significantly and positively correlated with its corresponding O_3_ concentration. The influence of the share of secondary industry on surface O_3_ in East and North China is significantly higher than that in other regions, and its regression coefficient exceeds 0.08. We also find that over 80% of the regional disposable income per capita is positively correlated with O_3_, with regression coefficients ranging from 0.07 to 0.36. In contrast, Guangdong, Shandong, and Northeast provinces show a significant negative correlation between disposable income per capita and O_3_, with regression coefficients, were below −0.02. The industrial dust emissions in Sichuan and Chongqing are significantly (*p* < 0.001) positively correlated with the corresponding O_3_ concentration with a regression coefficient > 0.52, while industrial dust emissions in cities located in East China are significantly (*p* < 0.01) negatively correlated with the corresponding O_3_ concentration with a regression coefficient ranging from −0.53 to −0.25, where O_3_ concentrations in cities located in eastern Jiangsu and Anhui provinces are more affected by the negative correlation of industrial dust emissions. The NOx emissions were significantly and positively correlated with O_3_ concentrations in Central China, East China, South China, Sichuan and Chongqing, and parts of Southwest and Northwest China (*p* < 0.05), with regression coefficients ranging from 0.60 to 1.26. There was a significant (*p* < 0.01) negative correlation between VOCs emissions and O_3_ concentrations in Hubei, Jiangxi, Zhejiang, Anhui, Jiangsu, Shanghai, Guangdong, Fujian, and Guangxi cities with regression coefficients ranging from −0.53 to −0.35.

**Figure 10:**
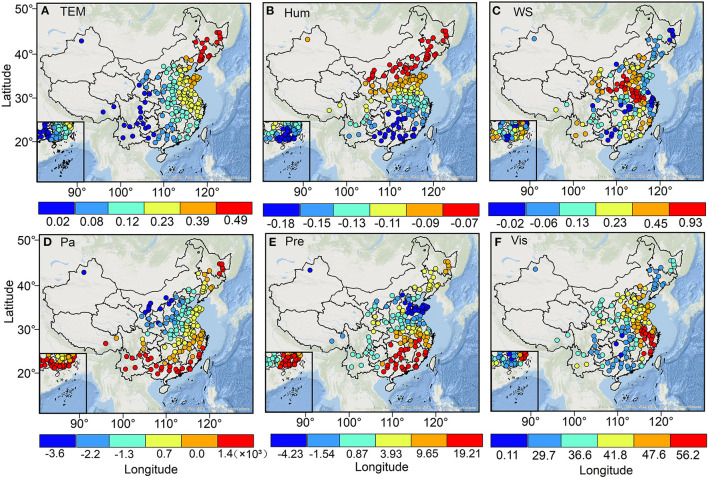
The spatial distribution of regression coefficients for the major meteorological factors of TEM **(A)**, Hum **(B)**, WS **(C)**, Pa **(D)**, Pre **(E)**, and Vis **(F)**.

Figure 10 shows the spatial differences in the effects of various meteorological factors on O_3_ concentration. It can be found that the temperature of cities in North, East, and Northeast China showed a significant (*p* < 0.05) positive correlation with O_3_ concentration, with regression coefficients ranging from 0.23 to 0.49. The relative humidity was negatively correlated with O_3_ concentration in all cities during the study period. Among them, cities in Heilongjiang, Jilin, Liaoning, Beijing, Tianjin, north-central Hebei, northwestern Shanxi, western Inner Mongolia, and northwestern Ningxia and northern Shaanxi showed a weak negative correlation between relative humidity and O_3_ concentration with a non-significant (*p* > 0.05) regression coefficient of < −0.07. In contrast, cities in southern Zhejiang, southern Anhui, Jiangxi, central Hubei, Hunan, Chongqing, Guizhou, Yunnan, and cities in Fujian, Guangdong, and Guangxi regions showed a significant (*p* < 0.01) strong negative correlation between relative humidity (Hum) and its corresponding O_3_ concentration with regression coefficients ranging from −0.18 to −0.15. Wind speed (WS) showed a significant (*p* < 0.05) negative correlation with O_3_ concentrations in Heilongjiang, Jilin, Liaoning, Guangxi, southern Henan, Hubei, eastern Shandong, Jiangsu, Shanghai, Zhejiang, Sichuan and Chongqing regions, and northern Shanxi, with regression coefficients ranging from −0.02 to −0.06. It is particularly noteworthy that cities in BTH, southwestern Shanxi, northern Henan, central Shaanxi, Ningxia, southern Gansu, western Shandong, and Anhui have a significant positive correlation between their wind speed and O_3_ concentration with regression coefficients >0.45. For air pressure, cities located in northern China showed a significant (*p* < 0.05) negative correlation between air pressure (Pa) and O_3_ concentration, with regression coefficients ranging from −3.6 × 10^−3^ to −1.3 × 10^−3^. Precipitation showed a significant (*p* < 0.05) positive correlation with O_3_ concentration in Heilongjiang, Jilin, South China, Guangxi, and Guangdong, with regression coefficients ranging from 3.93 to 19.21, while other regions showed negative correlations. Visibility was positively correlated with O_3_ concentration in all cities.

## 4. Discussion

### 4.1. Spatial distribution difference of O_3_ concentration

The results of the spatial and temporal pattern analysis of O_3_ concentrations show that East, Central, and North China are the regions with the highest growth of O_3_ concentrations in China from 2013 to 2018, which is mainly attributed to the huge amount of anthropogenic emissions. The areas of East, Central, and North China are one of the most densely populated and industrially developed regions in China, and the massive industrial activities, transportation, and human activities result in the emission of large amounts of O_3_ precursors. In contrast, Southwest and South China are the regions with the largest decreases in O_3_ concentrations in China. Previous studies have found that Southwest and Northwest China are located in high-latitude regions, and their corresponding atmospheric vertical exchange and photochemical reactions are stronger due to the special topography and intense solar radiation compared to inland regions, resulting in higher background values of O_3_ concentrations in these regions (9, 31). However, the extent of the influence of solar radiation on O_3_ in southwest and southern China is significantly weaker than the influence of anthropogenic emissions compared to the dramatic increase in O_3_ concentrations due to strong anthropogenic emissions in East, Central, and North China (32).

### 4.2. Spatial heterogeneity of O_3_ concentration drivers

There are strong spatial variations in the influence of different drivers on O_3_. Relative to lower population density regions, a larger population size implies more energy consumption and pollution emissions, meanwhile, it also further compresses the green area of cities, leading to a significant reduction in the ability of cities to mitigate air pollution, which better explains why the positive correlation between population size and O_3_ concentration is significantly higher in densely populated northern and eastern China than in other regions (21, 33). Previous studies have shown that industrial emissions are the predominant source of air pollution (34). Our study found that the share of secondary industry in GDP had a significant positive correlation with O_3_ concentration, especially in central China, and eastern China, where industrial production is dominant, and the contribution of urban industrial production to O_3_ concentration is stronger than in other regions. At the urban scale, the formation of O_3_ concentrations depends on the VOCs–NOx ratio (35). In general, the higher the NOx emissions in cities, the lower the VOCs–NOx ratio. For example, the formation of O_3_ in some cities located in Central and Northern China is often limited by VOCs (36, 37). In these cities, the reduction of VOCs emissions decreases the formation of O3, but the reduction of NOx emissions increases the formation of O_3_. This chemical reaction tends to depend on the amount of VOCs and NOx emissions; the larger the emissions the more intense their reaction and the larger the O_3_ emissions generated (38). In addition, industrial dust emissions indirectly affect solar radiation intensity by affecting atmospheric visibility, which further contributes to the O_3_ photochemical reaction rate (39).

Temperature is an important ambient condition for photochemical reactions, and higher temperatures can promote the rapid production of O_3_ concentration, therefore, temperature and O_3_ concentration are mostly positively correlated, especially in cities in Northern, Eastern, and Northeastern China where the solar temperature is higher in the warm season (40). The wind speed has a diffusion and transport effect on pollutants in the atmosphere. For example, O_3_ concentrations in cities in Northeast, South, Central, and East China, and Sichuan and Chongqing regions showed a significant (*p* < 0.05) negative correlation with wind speed. However, our results found a significant positive correlation between O_3_ concentration and wind speed in most cities located in the North China Plain. Li et al. (41) attributed the significant positive correlation between O_3_ concentration and wind speed in the North China Plain region to the influence of warm-season burning winds, especially from June to August each year, when the burning winds blow from the mountains to the northern and western parts of the North China Plain, bringing dry the hot air further leads to a higher temperature in the region, which accelerates the photochemical reaction of O_3_ production to some extent. Relative humidity has a negative correlation with O_3_ concentration. Previous studies have shown that water vapor can not only absorb and release energy through changes in the aqueous phase but also undergo internal reactions, especially when controlling for other influencing factors, higher relative humidity leads to higher water vapor saturation, resulting in easy removal of O_3_ and its precursors and lower O_3_ concentrations (42). In addition, water vapor can reduce solar ultraviolet radiation through extinction mechanisms, thus affecting photochemical reactions and O_3_ concentrations (43).

### 4.3. The O_3_ control policy implications

In summary, O_3_ pollution in China is gradually increasing, and more and more of China's population is exposed to high O_3_ concentration pollution. Scientific and effective reduction of O_3_ concentration exposure levels in China is crucial to reduce population exposure risks (44). Under these circumstances, this study proposes policy recommendations on how to reduce O_3_ concentrations in Chinese cities from the perspective of the drivers affecting the spatial distribution of O_3_ and epidemiology. For O_3_ pollution areas dominated by O_3_ precursors (e.g., NOx, VOCs, and CO), the authorities can ensure that their emissions comply with government regulations by optimizing the industrial structure and reducing the emissions of O_3_ precursors. Meanwhile, the governmental department should focus on the synergistic management of PM_2.5_ and O_3_ compound pollution. Research shows that NOx is not only an important precursor for O_3_ generation but also an important precursor for PM_2.5_ (45). Therefore, strengthening the NOx deep regulation and emission reduction is a key step to promote synergistic control. Furthermore, the O_3_ abatement measures in the future should pay attention to different seasonal O_3_ control measures and strengthen regional cooperation for O_3_ pollution prevention.

For O_3_ pollution regions dominated by meteorological factors, the department should forecast the variation of O_3_ concentration due to the change of meteorological factors promptly, meanwhile develop a detailed O_3_ pollution early warning program to reduce the risk of public exposure and explore a sustainable development path for O_3_ pollution management in China. From an epidemiological perspective, to protect public health and improve the status of O_3_ pollution, it is crucial to establish studies of health effects attributed to O_3_ exposure from a national perspective. In addition, it is important for relevant government departments to establish a mechanism to revise the National Ambient Air Quality Standards (NAAQS) for regulatory assessment and health risk prediction of future O_3_ air quality standards in China (46).

### 4.4. Research limitations and future prospects

Surface O_3_ distribution has strong spatial and temporal heterogeneity, and there are significant differences in O_3_ concentrations with time scales. This study only focused on the interannual spatial variability characteristics of O_3_ concentrations, neglecting the seasonal variability of O_3_ concentration changes. Furthermore, due to the lack of basic research data and inadequate research methods, this study only focused on the number of premature respiratory deaths attributed to O_3_ pollution in the assessment of health risks attributed to O_3_ pollution, neglecting the all-cause premature death group. Additionally, using the same exposure risk coefficient (β) may lead to spatial errors in the estimated health risks due to significant spatial differences in O_3_ exposure levels. For example, Wang et al. (21) estimated the population of premature deaths from respiratory diseases caused by O_3_ pollution between 2013 and 2017 in China using the method of Turner et al. (47), and their results found an average of 186,000 deaths from respiratory diseases due to O_3_ pollution during the study period. This is slightly lower compared to our findings. A primary reason for this is that our study and Wang et al. (21) used different exposure response coefficients and critical thresholds. In addition, the interpolation of O_3_ concentrations at large scales of pollution can also cause large errors in the assessment results. Therefore, in the future, we hope to conduct a detailed and comprehensive analysis of seasonal differences in O_3_ pollution and all-cause health risks in China by utilizing more detailed surface O_3_ monitoring data and meta-analysis methods. To provide a scientific basis for the improvement of O_3_ pollution in China.

## 5. Conclusions

In this study, we quantitatively investigated the spatial and temporal patterns, trends, population exposure risks, health risks, and drivers of surface ozone in China from 2013 to 2018. We observed the annual average O_3_ concentration of China increased significantly at a rate of change of 1.84 μg/m^3^/yr from 2013 to 2018 (*p* < 0.05, *R*^2^ = 0.561). The significant increase was mainly distributed in East China, Central China, and North China. Meanwhile, the growth rate of O_3_ concentration has a consistent and enhanced positive spatial autocorrelation (*p* < 0.05), and there are significant hot and cold spots areas. During the research period, there was an average of over 24,000 premature deaths from respiratory diseases attributed to O_3_ exposure in China from 2013 to 2018, and the growth rate fluctuated at 1,178 per year (*p* < 0.05). Spatially, there was a consistency in the spatial distribution of exposure risk and health risk of populations exposed to O_3_. The results of the multi-scale geographically weighted regression model reveal spatial differences in the effect of various factors on O_3_ concentration. The impact of the total population, disposable income, the share of secondary industry in GDP, and NOx emissions factors in eastern and northern regions are significantly greater than impacts in central and western regions. Meanwhile, we found that the effect of temperature on O_3_ concentration in some cities in the north, east, and northeast is significantly higher than that in other regions, and relative humidity has a significant (*p* < 0.01) strong negative correlation with O_3_ concentration in east, central, southwest and south China.

## Data availability statement

The datasets presented in this study can be found in online repositories. The names of the repository/repositories and accession number(s) can be found in the article/supplementary material.

## Author contributions

CH conceived the idea of the study and designed research, wrote the paper, discussed the results, and revised the manuscript. LZ conceived the idea of the study and design research, discussed the results, and revised the manuscript. XG involved in funding acquisition, resources, supervision, and analyzed the data. BL and JL discussed the results and revised the manuscript. QW analyzed the data, discussed the results, and revised the manuscript. All authors contributed to the article and approved the submitted version.
